# The State of the Art in Root System Architecture Image Analysis Using Artificial Intelligence: A Review

**DOI:** 10.34133/plantphenomics.0178

**Published:** 2024-04-18

**Authors:** Brandon J. Weihs, Deborah-Jo Heuschele, Zhou Tang, Larry M. York, Zhiwu Zhang, Zhanyou Xu

**Affiliations:** ^1^ United States Department of Agriculture–Agricultural Research Service–Plant Science Research, St. Paul, MN 55108, USA.; ^2^ Department of Agronomy and Plant Genetics, University of Minnesota, St. Paul, MN, 55108, USA.; ^3^ Department of Crop and Soil Sciences, Washington State University, Pullman, WA 99164, USA.; ^4^ Biosciences Division and Center for Bioenergy Innovation, Oak Ridge National Laboratory, Oak Ridge, TN 37830, USA.

## Abstract

Roots are essential for acquiring water and nutrients to sustain and support plant growth and anchorage. However, they have been studied less than the aboveground traits in phenotyping and plant breeding until recent decades. In modern times, root properties such as morphology and root system architecture (RSA) have been recognized as increasingly important traits for creating more and higher quality food in the “Second Green Revolution”. To address the paucity in RSA and other root research, new technologies are being investigated to fill the increasing demand to improve plants via root traits and overcome currently stagnated genetic progress in stable yields. Artificial intelligence (AI) is now a cutting-edge technology proving to be highly successful in many applications, such as crop science and genetic research to improve crop traits. A burgeoning field in crop science is the application of AI to high-resolution imagery in analyses that aim to answer questions related to crops and to better and more speedily breed desired plant traits such as RSA into new cultivars. This review is a synopsis concerning the origins, applications, challenges, and future directions of RSA research regarding image analyses using AI.

## Introduction

World food insecurity and malnourishment continue to drive agricultural breeding efforts to produce higher quality and increased quantities of crops with less land and fertilizer as human populations profoundly increase. The “First” Green Revolution emphasized genetic improvements through breeding for aboveground plant traits such as yield and standability while largely ignoring the below ground traits and concomitantly used new technologies such as synthetic chemical fertilizers, pesticides, and herbicides, updated heavy equipment for more automated agricultural tasks, and more efficient irrigation practices [1]. Plant breeding is currently on the cusp of the “Second Green Revolution”, which is described as the improvement of plants (especially root systems) through their abiotic stress tolerance and nutrient/water acquisition/usability and efficiency [2]. The First Green Revolution focused on high input systems and maximum yield potential, while the Second Green Revolution will increase yield potential in lower input systems. The new emphases on previously unstudied root traits such as their architecture, morphology, efficiencies related to water, nutrients, and tolerance to degraded (low fertility) or toxic (salt, acidity, etc.) soils have come to the forefront of research in hopes that the same gains achieved with breeding for aboveground traits can be realized by breeding for optimized root systems. The advent of computer-assisted techniques to study root system architecture (RSA), such as computer vision (CV) and artificial intelligence (AI) methods, including deep learning (DL), coupled with the increased processing ability of modern computers to deal with large amounts of data efficiently has ushered in a new era of RSA research. CV refers to the intentional application of user-defined algorithms to process digital imagery through methods such as thresholding pixels based on color or counting pixels. On the other hand, AI methods make use of the concept of training in order for computers to solve problems without user-defined algorithms. Here, we outline the importance of roots in agriculture and provide a framework to understand the applications and future directions for CV and AI in root research.

## Why Roots?

Plant roots serve many functions, such as the mechanical/structural support of the aboveground material, as acquisition organs for water and nutrients, as storage areas that support perennial growth, and as the symbiotic interfaces for relationships with other organisms such as nitrogen-fixing and other bacteria, mycorrhizal fungi, and other subterranean microbes. Plant roots also serve as a soil conservation apparatus where roots reinforce soil by increasing its tensile strength [3,4]. In the case of perennial roots, such as alfalfa, soil can be strengthened to depths over 6 m [5] and deposit more soil carbon at greater depths than many shallow-rooted plants (especially annuals) such as maize (*Zea mays* L.) and soybean (*Glycine max* L.). Therefore, breeding plants to have a specific root ideotype (or ideal phenotype) for a specific benefit(s) is advantageous [6]. Examples include breeding efforts that seek steep-angled roots for increased mobile resource acquisition, such as water and N for cereals [7], shallow-angled roots for immobile P, or deep root systems capable of increased C sequestration at greater depths resulting in improved soil structure, and water and nutrient retention [8]. Furthermore, the greater depth of plant root systems also gives plants more flexibility in drought times with the ability to reach or maintain contact with a declining water table [9]. Developing crops with root systems that can acquire more water and nutrients would directly improve food security and economic development in poor nations and would improve the sustainability of agriculture in rich nations by reducing reliance on intensive fertilization and irrigation [10].

**Fig. 1.**
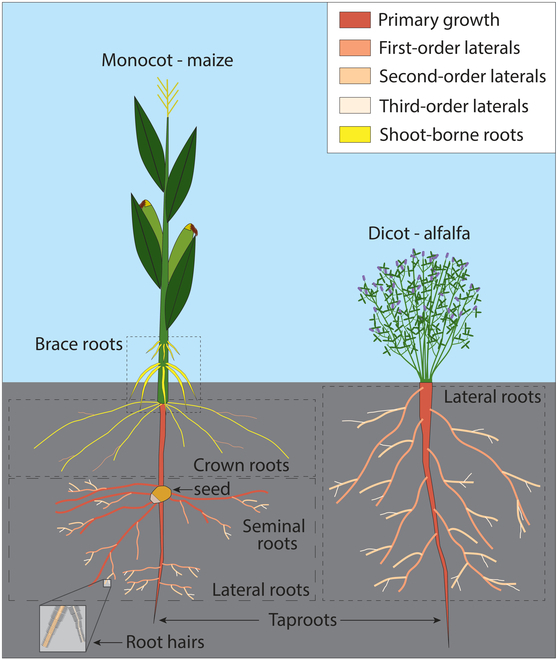
Monocotyledon (monocots) and dicotyledon (dicots) root systems. Root and shoot scales are exaggerated for detail. Taproots also called radicle and primary root in some literature (see [112] for detailed plant root system architectural taxonomy discussion).

Breeding efforts to produce root ideotypes with specific functions such as optimized nutrient or water acquisition and tolerance to stresses such as salt, drought, or acidic soils are conscribed by several root properties, including root morphology, root topology, root distribution, and RSA. Root morphology refers to the surface features of a single root and the characteristics of the epidermis, including root hairs, the pattern and appearance of daughter roots (primary, secondary, and tertiary lateral roots), the root cap, undulations of the root axis, cortical senescence, and root diameters [11]. Root topology refers to the way in which constituent roots of the root system are connected to each other in terms of their axes and branching. Topology is stable to deformation or rotation of the axes and can therefore be measured from excavated root systems. Root distribution is concerned with the presence of roots (not their orientations) in a positional gradient or grid, and typical studies involving distribution are interested in root biomass or length as a function of other qualities such as soil depth, distance from the stem, and position between neighboring plants [11]. RSA refers to the spatial configuration of the entire root system or large subsets of individual plants (the explicit geometry of root axes) and not its fine structural details, component parts, or singular elements (morphology and topology) such as root hairs. RSA is the spatiotemporal arrangement and the complex assemblage subunits of the root system that has functional significance and describes distinct aspects of the shape of root systems [11]. RSA is descriptive of the entire root system, such as multiple axes, and supersedes both topology and distribution in that both topology and distribution are known if architecture is known; however, neither topology nor distribution can be used to derive architecture (which is quite complex in mature plants). Figure 1 illustrates the differences in RSA between monocotyledons (monocots) and dicotyledons (dicots) using maize and alfalfa as examples; however, RSA varies considerably between species, among genotypes and phenotypes of specific species, within different parts of individual root systems, and through time as root systems mature and grow in complexity.

Several authors interested in RSA have commented that the largely unseen qualities of roots are a fertile, yet often overlooked [11–15], understudied or rare [16,17] research topic when it comes to producing phenotypes of crops with more desirable traits. Plant breeding has confounded the relationships between root traits and shoot/leaf traits [18] such as breeding for increased grain yield that resulted in increased stem and root lodging in wheat, barley, and oats [19]. Plants such as alfalfa, bred for aboveground biomass, may have ignored the importance of roots, their structure, and overall contribution to the function and success of the selected lines [14]. On the other hand, evidence exists that aboveground selection led to indirect selection on efficient root trait values [20]. While many breeding studies have seen correlations between root and leaf area or nutrient concentrations [21], other correlations between root traits such as root length and aboveground traits such as leaf area or nutrient content do not always exist [18]. In general, root mass and shoot mass are expected to be correlated because of plant allometry, but this pattern seems to be disrupted because of domestication and root plasticity from environmental stresses.

Observing, quantifying, and interpreting the spatiotemporal root structure and growth habits of the belowground portion of plants have been a major limitation to breeding efforts and high-throughput phenotyping that focuses on improving root traits [15]. Roots by their very nature are obscured by soil or opaque growing mediums that can cause high amounts of background noise in images or scans of in situ roots and root systems. In addition, some plants like alfalfa can have root systems over 15 m in depth [22], and the scale of the root system becomes a limiting factor to studies interested in capturing images of that system in its entirety.

## Root Acquisition

Methods of root material acquisition for RSA measurements fall along a continuum based on the degree of destruction and removal of roots from the growth environment (destructive to nondestructive). Root harvesting, which is the physical removal of large portions or nearly entire root systems, is the most invasive and destructive method of root acquisition and allows for 3-dimensional (3D) visualizations after the removed materials are washed of soil or growth media and imaged with a variety of possible sensors [most commonly flatbed scanners and red–green–blue (RGB) cameras; Table]. Scanning washed roots on flatbed scanners with transparency units while suspended in water may be the most standard method, but has limitations.

**Table.** Summary of common methods for plant culture and root phenotyping

**Plant growth location**	**Root phenotyping method**	**Destructive**
Laboratory	Growth and luminescence observatory for roots (GLO-roots)	No
	Rhizophonics (liquid-based)	No
Laboratory/greenhouse hybrid	Tomographic (x-ray, PET, MRI, etc.) (3D image)	No
	Rhizoslides (paper-based)	No
	Rhizotrons (preinstalled flat surfaced root windows)	No
	Clear pot method (transparent pots filled with soil)	No
Greenhouse	Rhizoboxes	No
	Washing roots from pots	Yes
	Root towers	No
Greenhouse/field hybrid	Root crown excavations (or shovelomics)	Yes
Field	Soil coring	Yes
	Trench excavations	Yes
	Rhizolysimeters	Yes
	Minirhizotrons (installed after plant growth via coring)	Yes

Oft-cited methods of root harvesting are shovelomics [23,24], trenching (pioneered by [25]) [18], and coring [26]. These methods are labor-intensive and shovelomics, for example, requires destruction of the growth environment and removal of the sample plants by removing the root crown. Trenching is less destructive in that the plant and its root system are exposed but not removed. Coring, while still destructive to individual roots, does not kill the sample plants, whereas shovelomics certainly does (unless transplanting occurs) and trenching severely perturbs the growth environment and exposed roots. Investigations that use shovelomics and trenching are limited in scope to root crowns and shallow portions of root systems and do not allow incremental growth to be studied as with nondestructive methods. However, shovelomics and trenching do allow for portions of plant root systems to be analyzed with their architecture, morphology, topology, and distributions intact, as opposed to limited sections of roots with coring with the positive caveat of greater sample depths than shovelomics or trenching achieve. Although shovelomics offers more complete viewing of root crowns, removing large portions of the root system via shovelomics from the growing medium and subsequent washing of the roots may result in the loss of fine materials such as root hairs and other fine root materials. Another issue with removing plants from the substrate is that the in situ orientations, especially of lateral roots, are likely to deviate in the absence of the buttressing effects of soil or growing medium they originated from. In contrast, nondestructive techniques for image acquisitions do not require plant roots to be removed from their growth environment.

Nondestructive techniques can be in either noninvasive or minimally invasive categories, where the former involves data/image acquisitions that do not damage plants and the latter acquires data via small perturbations of growth or minorly damages plants [27]. There are several advantages to some nondestructive imaging techniques such as the ability to conduct time-series analyses (4D) and to observe growth habits without disturbing (or minimally disturbing) the plant. 2D or (2D + time series) methods such as rhizotrons or minirhizotrons allow time-series data to be collected, which allows for incremental growth, growth habits, and other underground growth activity such as nodulation and mycorrhizal activity to be observed [28]. While noninvasive, field direct 3D techniques such as ground-penetrating radar measurements of fine root materials and root hairs are not possible because of the technology's lack of resolution [29]. However, the use of other sensors such as electromagnetic resistance, magnetic resonance imaging (MRI) [30], positron emission tomography (PET) [27], x-ray computed tomography (x-ray CT) [31], and neutron tomography (NT) [32] have been successful in greenhouse and laboratory environments. Nevertheless, nondestructive methods fail to sample portions of a plant’s root system due to the fine nature of some root material or root hairs, root water content, the presence of para- and ferromagnetic background materials that interfere with scans, because of the opacity of soil, or the limited area of the soil and root interaction that are sampled. In addition, roots often grow along the path of least resistance, so it has been suggested that roots grow along windows, possibly skewing aberrance and distribution data. Because of the difficulties with nondestructive techniques, a combined approach that involves both the removal of plants from their growing substrate and AI techniques that analyze root system images is growing in use, where images of root systems acquired by shovelomics are analyzed with the aid of computers using machine learning (ML) technologies These techniques have been applied to several major agricultural crops and plant types in studies concerning roots and RSA, such as alfalfa [14,17,33], apple (*Malus domestica*) [34], cotton (*Gossypium herbaceum* L.) [35], maize [36,37], millet (*Setaria italica*) [38], rice (*Oryza sativa* L.) [39,40], and soybean [16].

## Root Image Analysis Using AI and ML

A strength of AI and ML techniques is the in silico ability to detect, classify, quantify, and predict a multitude of features and patterns from disparate data in ways and amounts that are not possible by unaided humans. In addition, as ML techniques grow in popularity, their flexibility and scalability are propelling their applications into formerly unrelated fields and topics. ML applications can be found in many aspects of society with recognition and predictive analytics, chatbots, bioinformatics, identification/detection software, and plant genotyping/phenotyping [41].

Recent advances in technology, reductions of costs (imaging/sensor platforms), and progress in the subfields of AI are propelling plant breeding efforts forward through root image analyses of crops allowing for faster, high-resolution sampling and analysis of plant root phenotypic data [16]. Digital, automated techniques using CV and DL are quickly becoming the standard [42] and are considered critical to improving plant phenomics [16,41]. Over the past few years, software such as RootPainter [a popular graphical user interface (GUI) DL segmentation and annotation for training tool] [43] and RhizoVision Explorer (hardware and software for root measurements with more than 9,000 downloads to date) [44,45] have emerged as the preferred software for root measurements.

### What are AI, ML, and DL?

AI is the use of computers to mimic human intelligence via methods and techniques involving but not limited to if-then rules, logic, decision trees, and ML. ML and DL are subsets of AI technology. ML includes a group of computerized modeling techniques that can learn patterns and make decisions automatically without following directly programmed parameters [41]. A strength of ML is the ability to “learn” the inherent structures, commonalities, or differences in the data as the algorithm analyzes data allowing the model to explain or classify new or known phenomena and generalize trends and patterns [41]. DL moves beyond ML in that its methods involve representation learning that creates simple nonlinear modules that transform the representations at increasingly higher and more abstract levels that allow (with enough transformations) the learning of complex functions [46]. LeCun et al. [46] describe DL representations of image data (arrays of pixels) as layers of features (such as edges, motifs, parts of objects, etc.) learned from data using general purpose learning procedures independent of, and not implemented within the model by, human engineering (the model teaches/trains itself). Within DL are the subclasses of convolutional neural networks (CNNs) and recurrent neural networks (RNNs). CNNs are specifically designed for image data [47,48], which are 2D data structures [matrices of pixel values (picture elements)] [49] that contain visual sensory information, and are mainly applied to tasks related to CV, while RNNs explicitly analyze sequential data structures such as time-series 2D (images) and 3D (video) data [50]. CNNs are particularly well suited for their implementation into RSA research because of their improved ability to learn autonomously over previous ML techniques [35], but, specifically, CNNs can handle tasks involving data with spatial relationships of imagery where pixel values are immutable, i.e., do not change because images are static and not time lapse where spatially fixed pixel values change as a function time [50]. RNNs are capable of sequential pattern learning to capture/model time dependencies [51] that could make them applicable to change detection tasks like incremental growth and/or growth habits of roots using time-series data such as video or time-lapse photography, although at the time of this review’s submission, the authors are unaware of any research that utilizes this technique for RSA. For studies of RSA, the most common problems for use of AI are for image segmentation to identify root objects from complex backgrounds and for classification using features (trait measurements) typically extracted using conventional CV methods, such as root length and diameter.

CNN architectures represent DL-based approaches of complex pattern recognition tasks [52] dealing with spatial data and function in a feed-forward manner using filters and pooling layers. The basic architectural components of a CNN are convolutional layers, pooling layers, and fully connected layers. Convolutional layers involve linear operations that multiply a 2D array (called filters or kernels) of weights to the input image [48], and the role of convolutional layers “is to detect local conjunctions of features from the previous layers” [46], which outputs feature maps that represent specific features in the input image. Pooling layers function to downsample feature maps in 2 common ways, which, according to Brownlee [48], are (a) average pooling that summarize the average presence of a feature and (b) max pooling that summarize the most activated presence of a feature. Fully connected layers connect convolutional layers and pooling layers and reach a classification decision by connecting the input layers to the output layers.

The use of ML to analyze RSA can be traced back through the lineage of earlier research regarding CNNs and image classification, starting with LeCun et al. [53], who are credited with one of the earliest uses of a CNN method (LeNet-5) that they successfully used to recognize handwritten fonts. Later, Krizhevsky et al. [54] created AlexNet, an improved CNN-based method of automatic image classification that added rectified linear units to improve speed (6 times faster than CNNs using the tanh function), allowed for multi-graphics processing unit training, overlapping pooling [pooling unit spacing (*s*) < neighborhood size (*z*)] [54], and data augmentation (creating additional data using transformations on existing original data) [55] and dropout (randomized temporary removal of hidden or visible units in a neural network) [56] to reduce overfitting (failure of a model to describe underlying distributions instead of noise or variance in data) [57]. Another advancement to CNN technology included models that conduct semantic segmentation that involved labeling each pixel of an image with the class of the object or image region it belongs to [58]. This improvement was originally made to fully convolutional network models by simplifying and increasing the speed of learning to the inference of fine structures recovered, ability to separate closely interacting objects, and robustness to occluding objects [58]. Other advancements to sematic segmentation include the addition of scaling layers and filters that apply high-level learned behavior leading to better precision with a major reduction of training data (U-Net) [59], pixel-level feature to global pyramid pooling (Pyramid Scene Parsing Network) [60], capturing contextual information at multiple scales and decoder modules to recover object boundaries (DeepLab3+) [61], and differentiation of same-object-class contextual pixels from the different-object-class contextual pixels (object-contextual representation) [62]. Currently, YOLO9000 (You Only Look Once 9000), an object detection algorithm using CNNs [63], and YOLACT (You Only Look At Coefficients), a fully convolutional model for real-time instance segmentation [64], are utilized to improve speed through parallel subtasks.

In contrast, RNNs are well suited for tasks that involve temporal data with sequential inputs such as speech and language, are very good at predicting the next characters or words in a sentence, and can be used for translating from one language to another [46]. In the case of image analysis, RNNs can be paired with CNNs to create image content descriptions or annotations. A main strength of RNNs is that their architectures function by feeding results back into the network that allows the model to “remember” information and apply it to the next series of data to process, such as root dynamic development pictures/videos through time. In this way, modern RNNs that use long short-term memory use special hidden units called memory cells [46] that are able to make inferences about what is happening within a video or time-lapse photo series.

### The learning process and model objectives of ML

**Fig. 2.**
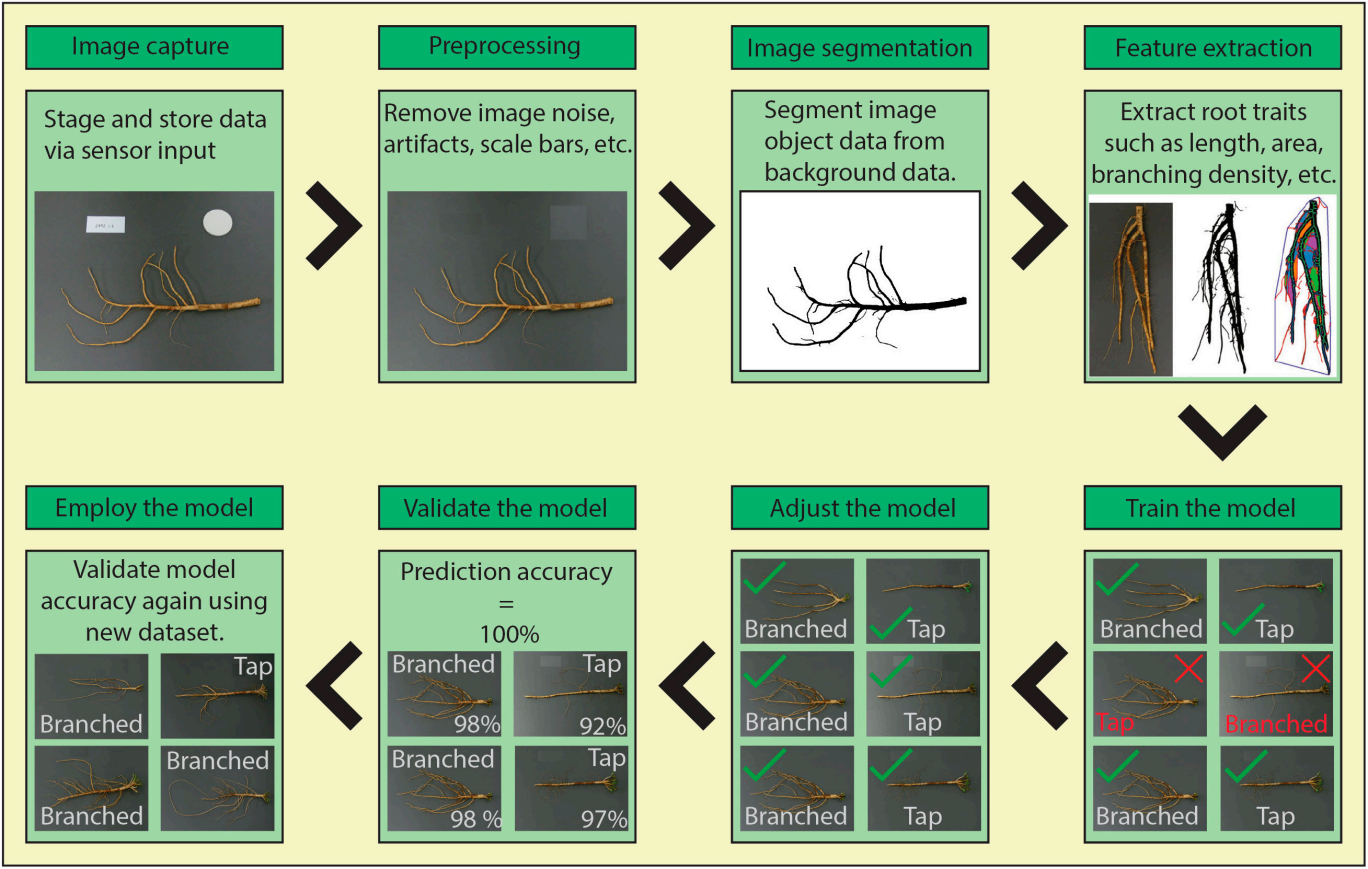
The ML for RSA research process (simplified) using alfalfa root images. The general steps include image capture, preprocessing, segmentation/feature extractions, model training, model adjustments, model validation, and model employment for identification.

In general, ML for RSA research is a multistep process that is as follows: (a) image capture, (b) preprocessing, (c) image segmentation, (d) feature extraction, (e) train the model, (f) model adjustment, (g) model validation, and (h) model employment for identification and detection, classification, and quantification (Fig. 2). Workflows (and partial workflows) similar to those shown in Fig. 2 are reported in Perez-Sanz et al. [49], Mattupalli et al. [65], Falk et al. [16], Narisetti et al. [37], and Xu et al. [17].

Image capturing involves many different spatiotemporal techniques (2D, 3D, and 4D) and sensor–platform combinations and has been reviewed extensively by Atkinson et al. [42] (a review focusing on RSA sensor/platforms). Once collected, image data typically require preprocessing steps that include the removal of unwanted artifacts by filtering out image noise and/or cropping. Of course, efforts to produce the highest quality data (clear, sharp, and well-lit/uniform lighting) that subsequently require minimal preprocessing are ideal but not always realized in all studies. Multichannel images may need to be converted to binary images (black and white) or masks for classification tasks. However, some models require multichannel data, such as classifying for particular signatures, like root properties, diseased roots, or root nodules that have unique RGB values. The preprocessing steps of ML are an effort to “concentrate information” [41] and thereby improve the signal-to-noise ratio by reducing unwanted and/or conflicting image information. In terms of hardware for imaging-based root phenotyping, there is a prevalence of diverse acquisition pipelines and a general lack of standardization that Falk et al. [16] describe as imperative to scaling plant phenotyping methods; however, there are many (often freeware) software available for digital analysis and extraction of root traits that can be used for AI model inputs. A comprehensive list and description of each software package can be found at quantitative-plant.org.

Next, model training is performed (Fig. 2). If the model is supervised, training involves using a large portion (~80%) of the labeled image dataset to train the model for correctly classifying image data and then make connections (map) between the input dataset and the output labels. Supervised learning models are typically time-consuming and costly processes because the images need to be annotated [52]. Self-supervised/unsupervised models, conversely, do not require annotated data to train the model making the method appealing to researchers interested in foregoing image annotation tasks [46]. Instead, the model is trained by imputing a dataset with no labels and forcing the algorithm to create classes based on the data. The training phase of image processing models is regularly being created, tested, and augmented. Hybridized learning models that incorporate semisupervised learning with “small”, “few”, or “short” learning methods [66] that do not require massive amounts of labeled training data are one possible solution to the current bottlenecks. In addition, lowering complexity and increasing the efficiency of model implementation will benefit interactions between signal processing and ML through deep neural network compression that potentially allows more efficient handling [52].

Model adjustment (Fig. 2) is done by tuning the model parameters to account for errors from misclassification, overfitting, and bounding box misalignments in the classification process. These errors are often calculated using a confusion matrix that quantifies the numbers of true positives, true negatives, false positives, and false negatives. Model performance is quantified by raw confusion matrix values and/or using metrics calculated from them that include the dice similarity coefficient (DSC), intersection over union (IoU), F scores (F_1_ and F_2_), sensitivity, specificity, and accuracy [17]. Loss functions such as DSC quantify the agreement between the ground truth (manual segmentations/annotations) and model outputs, which can then be used to iteratively weight the model’s parameters to improve its performance [64,67]. In the case of some root image data (such as minirhizotrons), class imbalances can be deleterious to model performance [67]. Problems arise from class imbalances when very small portions of the image foreground data (root pixels) and high accuracy scores (the ratio of correctly identified image pixels to all classified pixels) may reflect a model’s ability to correctly classify the background and not the foreground pixels (roots) [68]; therefore, DSC, IoU, sensitivity, and specificity metrics are generally used for RSA model quality assessment [69]. Several iterations of model adjustments are typically necessary that involve tuning/calibrating the model parameters to account for and remove errors such as overfitting/underfitting and/or model biases.

Finally, cross-validation, usually 5- or 10-fold cross, is used to adequately adjust the model to produce results with least amount of variation between the training and testing data (Fig. 2). This adjustment will allow the model to be accurately deployed on new datasets for detection, classification, quantification, and/or prediction tasks.

### ML challenges

There are multiple classes of challenges with varying levels of commonality concerning the implementation of ML models. The commonplace challenges of image quality, dataset sample sizes, training/testing class balance, overfitting, and model tuning are faced by nearly every researcher that performs ML image analyses. Challenges such as image label errors, lack of model results “explainability”, and adversarial attacks are less commonly faced but often come with more severe outcomes/consequences. Challenges related to groups of images (such as training data) for image analysis are among the most prevalent and typically encountered in CV [55]. First is large image variation between application and training models where images with similar content are inconsistently illuminated, have occluded content, have variable backgrounds or viewpoints, or are shown at different scales (domain shifts accounted for with training data). Another is image sample sizes or class imbalances where one or more classes have variable numbers of, or too few, sample images used for training. These types of variation can be mediated during image capture with proper planning or with the use of synthetic data as demonstrated by Wang and Cao [70]. Domain bias/shifts are challenges, and the resultant model overfitting errors occur from generalization from datasets (and image styles) not seen in training. One solution to domain shift problems is to fine-tune pretrained networks on new datasets [71] before implementing the model on a large scale. In addition, the issue of overparameterization is a new challenge brought about by DL that results from a model having a large set of learnable parameters yet has insufficient training data to overcome generalization problems expressed as overfitting [72]. The fact that overparameterization leads to decreased consistency of a learned hypothesis and is almost certain to occur has been demonstrated by some authors such as Seidenthal et al. [73] who investigated (in part) the effects of hyperparameter tuning. Image augmentation is a viable technique that can help prevent (in part) the issues of overparameterization, domain shifts, image sample sizes, and image variation [55,74,75]. In general, image augmentation involves applying an algorithm to an existing image that creates additional image data from the initial image with a variety of techniques, thereby increasing the amount of training data. Some examples of image augmentation include (but are not limited to) adding Gaussian noise, cropping, flipping, rotating, random erasing, or changing the scale of an image [55]. However, augmented image data are not a suitable replacement for actual genuine/original data because the augmented data are a derivative of the original sample image, which can cause a model to be sensitive to the underlying, true data no matter how manipulated it is via augmentation. Unfortunately, the collection of original (unique) data involves some of the more time-consuming tasks in the image analysis research process, and there is no equal replacement for original data.

Acceptance of the use of DL models is challenged as to how they come to the prediction results. These trust issues and/or lack of justification to use a model or use its results [76] are because DL is often described as “black box” and tends to lack “explainability” [52]. To help alleviate this problem, researchers are investigating a wide variety of methods and techniques that both access model accuracies and elucidate the underpinnings of model results by providing/pairing “explanations” to DL models. The vast number of methods used to assess model accuracy is beyond the scope of this work; however, a detailed review with examples can be found in Marcinkevičs and Vogt [77] and a field guide on the subject by Ras et al. [76]. According to Ras et al. [76], explanations fall into 2 general categories: (a) explanations that give insight into model training and generalization and (b) explanations that give insight into model predictions. For example, an explanation for image data may be a saliency map or heatmap, which is a depiction of the regions of interest that the model used to determine the network’s prediction. Heatmap-paired models offer increased trustworthiness over competing models with similar accuracy scores because a heatmap-paired model is more consistent with human experience [76].

Other regrettable challenges to DL models are knowing the provenance and/or provenience of an image and its authenticity, purity (unperturbed/unmanipulated data), or label quality. For example, images from public databases or secondhand data can have poor labels and/or be altered and therefore be of questionable integrity and validity. Northcutt et al. [78] investigated label errors contained within the most commonly used benchmarking datasets used for CV with a label confidence algorithm called confident learning [79]. They found “numerous and widespread” label errors in 10 prominent datasets and estimated an average of 3.3% errors across these validation datasets [MNIST (0.15% = 15 images), CIFAR-10 (0.54% = 54 images), Caltech-256 (1.54% = 458 images), CIFAR-100 (5.85% = 585 images), ImageNet (5.83% = 2,916 images), and QuickDraw (10.12% = 5,105,386 images)] [78]. In the case of QuickDraw, evaluating the errors of over 5 million mislabeled images is not feasible/tenable for most researchers, and the fact that this is a benchmark dataset used for training or validation poses a major problem for researchers interested in achieving the highest accuracies possible for their studies. The somewhat new challenge of introduced image errors with malicious intent are defined as adversarial attacks [80,81] that are perturbations, sometimes undetectable by humans (Gestalt law-based perception [82]), introduced to formerly “clean” images that cause neural network classifiers to completely change their predictions and erroneously predict the same image content with surprisingly high confidence [83]. This situation poses a threat to any researcher that relies on publicly available or secondhand image data to train their models where the trustworthiness and/or validity of those data is questionable. There do not appear to be many adversarial attack examples related to plant breeding efforts at this time; however, any image can be perturbed with nefarious intent to interfere with ML model results, and this poses a future problem for RSA researchers. This is because RSA investigations relying on DL methods and/or “mega” datasets composed of millions of annotated root images will require supercomputing power to process them and researchers will not likely be able to easily produce their own (pure and trusted) data (millions of pure images); therefore, secondhand image data with unknown authenticity will need to be utilized and/or reckoned with if its perturbed via adversarial attacks. In addition, as RSA research utilizing benchmark datasets that contain all types of subject matter (not restricted to root images) increases, the likelihood of perturbed imagery problems will also increase, so in this sense, the threat of adversarial attacks to RSA research results is increased even if the perpetrators of such image tainting are not directly targeting plant scientists or their results (the damage to RSA research will likely be collateral). Furthermore and as discussed with label errors in large benchmarking datasets, images containing adversarial attacks may not be easily identified by hand because of sheer volumes of datasets (millions of images) and/or because the introduced errors are not perceptible by humans. Methods to circumvent adversarial attacks include training a model with input data containing adversarial example imagery (called a “brute-force” strategy) to preempt the issue, modifying the network to detect image perturbations, and network add-ons that rectify perturbed images [80]. Some authors, such as Bai et al. [84], describe the need for model robustness to adversarial attacks as vital to safety and for maintaining high-quality results free from unwanted errors.

### Case studies of AI applied to root structure analysis

There are many studies that use a variety of image segmentation and analysis software to perform RSA research. In plant research, data pipelines involving AI have been utilized for identification and detection, classification, and quantification of a plethora of plant traits. To date, the fledgling use of AI to investigate RSA has markedly improved and hastened RSA research (of some authors) by replacing/suppressing the mostly manual time- and labor-intensive methods such as manual root segmentation with automated feature extraction and AI-based trait analyses. Some platforms and sensors such as tomographic scanners [i.e., electrical resistivity tomography (ERT), MRI, NT, PET, and x-ray CT], and other 3D imaging approaches are exemptions to the shifting paradigm of RSA analysis because of low-throughput and high cost, which is preventative to large-scale integration of the techniques into genetic research [16].

**Fig. 3.**
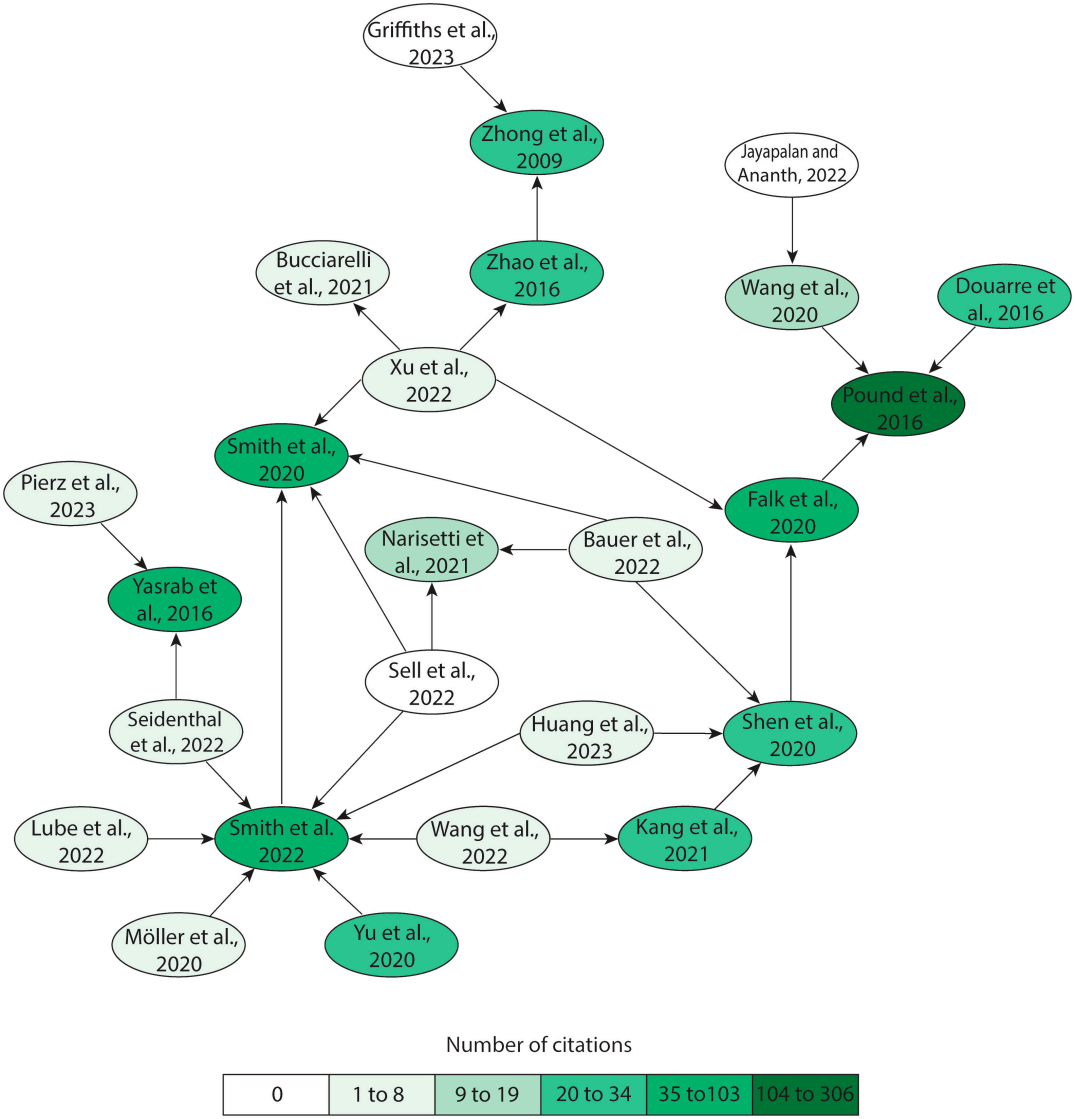
Network diagram of cited AI research literature involving root imaging. Citation numbers were acquired from Google Scholar citation database. Classification breaks were calculated using the Jenks natural breaks method. Arrows indicate the citation directions, and color indicates the frequency of citations. Although a meta-analysis was not performed, the authors used key word search terms: “root structure architecture”, AI-artificial intelligence”, and “image analysis”. The terms were inputted into multiple databases (Scopus and Google Scholar). Then online tools were used to find connections between researches and via cited references in acquired literature.

At present, there are several studies that use ML image analysis models on root properties and/or RSAs of a variety of plant species (Fig. 3).

Zhong et al. [85] appears to be the first research group to apply AI techniques to roots in an effort to classify RSA. They achieved this by harvesting maize roots via the shovelomics method and then performed laboratory-derived image captures on the sample roots with monochrome cameras (2,215 roots in total). The model they used for their extracted-feature-data-based classification was an SVM (support vector machine) that correctly classified 99.95% of the maize roots of 235 genotypes. Pound et al. [86] is another group that pairs CV with root structure analysis to investigate winter wheat root tips (*Triticum aestivum* L.) using 2,500 laboratory-derived images acquired from a digital single-lens reflex (DSLR) camera (RGB). The CNN used to classify whether root tips were present in an image (root tip present or no root tip present) had an accuracy of 98.4% using the Caffe DL library, and the problem of overfitting is stated to have been avoided by randomly deactivating a percentage of fully connected neurons during each iteration of training (50% dropout rate). The authors predicted a paradigm shift from manual trait/feature acquisitions to image-based phenotyping using DL approaches in the future, which is currently coming to fruition. This shift (from manual to more automated or in silico techniques) in methods can be seen as a milestone in RSA analysis research (a class of analysis we discuss below as phase I). Another group (Douarre et al. [36]) focused on maize roots (and simulated roots) from laboratory-derived x-ray tomography images and simulated root pixels using a pretrained (on ImageNet) SVM model [36]. The authors demonstrated that a transfer learning approach produces adequate results on simulated or real roots when the soil-root contrast is very low [36]. Zhao et al. [60] investigated that roots from 16 European pea (*Pisum sativum* L.) cultivars from laboratory-derived photo scanner (grayscale) images of greenhouse grown plants were first analyzed with WinRhizo 2012b Pro to obtain feature data (such as primary root diameter, length, surface area, etc.) and then subsequently analyzed using a combination of radial basis function SVM and random forest (RF) models resulting in the accurate classification of 86% of genotype pairs. The combination of radial basis function SVM and RF models could overcome problems related to applying ML methods to images with low signal-to-noise ratios [60]. As a solution to image segmentation and feature extraction challenges such as nonautomatic segmenting and root type classification and the inability to locate key features used to derive root geometry, a novel image analysis approach was developed, called RootNav 2.0, driven by a multitask CNN architecture [87]. Arabidopsis (*Arabidopsis thaliana*), rapeseed (*Brassica napus*), and wheat seedlings from 3,360 laboratory-derived images were acquired from a DSLR camera (RGB). The results indicate that the improved and now fully automated method achieved comparable accuracy to the original RootNav tool (semiautomated) but with a 10-fold increase in speed and reduced training data requirements with image domain shifts [87]. In addition, RootNav 2.0 is capable of segmenting high-resolution images, classifying root orders, and provides a strong foundation that users can derive common architectural traits [87]. Another group (Falk et al. [16]) developed a seamless end-to-end pipeline that investigated soybean roots with 12,264 laboratory-derived images captured from a DSLR camera (RGB). They used a convolutional autoencoder CNN framework for root segmentation and used advance root image analysis (ARIA) to extract root traits from segmented images and validated the outputs using manual assessments that produced a high-quality, cost-effective, mobile, phenotyping platform and high-throughput pipeline capable of quickly processing and analyzing thousands of plants [16]. The above case studies exemplify some of the many advances toward the current state of the art in RSA analyses using images, which began with progressions related to the speed of processing [also part in parcel to advances in graphics processing unit performance at the time AlexNet was developed (3-GB maximum memory)], the major shift away from manual segmentation and measurements of traits to more automated methods (automated trait/feature extractions, phase I), and some of the first studies that investigated unharvested plant roots still in soil/growing mediums (x-ray tomography, etc.).

Image segmentation is an important preprocessing step in more recent RSA image analyses studies and was an important avenue of inquiry that helped advance the field to its current state. In many of the investigations featured below, the move toward automated image segmentation using ML methods focused on the advantages of both speed and accuracy with automated methods to segment/detect roots in images from a variety of sensor types. Smith et al. [67] demonstrated the feasibility of segmenting roots in soil images with a U-Net-based CNN architecture, the ability of small research groups in need of self-generated custom-labeled datasets to use DL approaches, and replacement of the manual line-intersect method by evaluating chicory (*Cichorium intybus* L.) roots using 892 laboratory/field hybrid (outdoor root tower) images acquired from rhizotrons located in root towers (RGB). Their results indicate that they achieved an F_1_ score of 0.7 when comparing automated segmentation to manual annotations (automated segmentation outperformed the manual method). Automated approaches to identifying anatomical traits in microscopy root cross-sections by investigating rice roots were also focused on by Wang et al. [40]. They used and compared the Faster R-CNN and Mask R-CNN ML models producing an IoU of 0.95 for both root and stele objects, showing that the Faster R-CNN models can accurately detect and predict anatomical objects in images while concurrently producing more accurate results with smaller amounts training data than the competing model. Another study [88] interested in automatic root detection and segmentation using ML investigated switchgrass roots (*Panicum virgatum* L.) using 30 field-derived images obtained from minirhizotron scanners (RGB). They tested several ML models including multiple instance learning (MIL) models (MI-ACE, miSVM, and MIForests) and non-MIL models (SVM and RF), compared the models on the basis of receiver operator characteristic curves, and found that the miSVM method outperformed the competing models with a higher mean sensitivity (recall/true-positive rate) at the same false-positive rate and the MI-ACE model was a close second. Overall, they concluded that MIL methods can reduce the burden of image labeling and substantially improve imaging bottlenecks of root studies relying on minirhizotron techniques [88]. Another group [89] was also interested in automated segmentation of in situ root systems with ML and investigated cotton roots using 200 field-derived images acquired from a minirhizotron scanner (RGB). Their model was an improved version (via upsampling) of the DeepLabv3+ segmentation model [90] that outperformed both manual and U-Net segmentation methods with cross-fold validation of 0.9702, 0.9847, and 0.9773 for precision, recall, and F_1_ score, respectively [89]. The authors noted that their novel automated method more accurately and quickly segments root systems in complex soil environments while also outperforming antiquated manual segmentation techniques. They also comment that to prevent the network from overfitting, the weight attenuation was set to 1 × 10^−6^. Kang et al. [91] also experimented with the DeepLabv3+ model with the use of cotton roots using 150 laboratory-derived images (expanded to 263 images via augmentation) acquired from an in situ photo scanner (RGB) apparatus. Their AM-DeepLabv3+ semantic segmentation model was improved by adding an attention mechanism that assigned more weight to the pixel points of fine roots and root hairs. The quality metrics of their improved model, which outperformed U-Net, SegNet, and the traditional DeepLabv3+ models, were 0.9971, 0.9984, 0.9937, and 0.9875 for precision, recall, F_1_ score, and IoU, respectively [competing models were U-Net = 0.8923 (IoU) and 0.8919 (F_1_), SegNet = 0.9564 (IoU) and 0.9489 (F_1_), and traditional DeepLabv3+ = 0.9798 (IoU) and 0.9773(F_1_)]. They also noted that their proposed model can accurately distinguish cotton root systems from complex soil background with good segmentation effect [91].

Möller et al. [92] experimented with several different CNN architectures in an effort to challenge competing models with their proposed improved model and also to reconstruct the complete RSA by focusing on extracting the main root. Their analysis used data from Arabidopsis roots using 2,475 laboratory-derived video images (RGB). The ML models they used were SegNet and U-Net CNNs. Their championed model, U-Net with VGG16 structure, achieved DSC scores for “image complete”, leaf region, and nonleaf region of 0.911, 0.879, and 0.926, respectively.

Alfalfa seedling (14-d-old) RSA and trait heritability were investigated using RF and gradient boosting machine ML algorithms by Bucciarelli et al. [14]. Their results discuss the relative importance of secondary roots longer than 2.5 cm, indicating that using alfalfa seedlings as young as 14 d can be used for root trait selections, thereby hastening conventional phenotypic selection by 20 weeks (a rapid selection process allowing for 2 cycles of selection in a single year).

A modular, mobile system to continuously monitor seed germination and root growth called MultipleXLab was developed by Lube et al. [93]. The system utilized time-lapse laboratory-derived images of Arabidopsis and tomato roots (*Solanum lycopersicum*) acquired from a DSLR camera (RGB) and a “customized minirhizotron scanner” that consisted of transparent polystyrene square petri dishes. Two separate models (SeedNet and RootNet) were developed and subsequently used for this research that achieved F_1_ scores (harmonic mean of precision and sensitivity) of 0.8048 and 0.7395, respectively. The noninvasive, high-throughput system allows for high-precision evaluation of germination index and hourly growth rate between mutants and is a mobile alternative to high-end imaging platforms and stationary growth chambers [93].

The root structure architecture (branch-style, taproot-style, and taproot-branch roots) of mature, field-grown, alfalfa roots from 617 laboratory-derived images (and an additional 6,170 created augmented images) captured from an RGB DSLR camera was investigated by the increasingly popular pairing of RootPainter segmentation and RhizoVision Explorer feature extraction methods by Xu et al. [17]. Multiple ML models were tested include *k*-means, PAM (partition around medoids), RF, naïve Bayes, neuralnet, and Keras/TensorFlow resulting in the decision-tree-based RF model having the highest balanced accuracy values of 0.843, 0.852, and 0.703 for branched, taproot, and intermediate taproot-branch root types, respectively. In addition, they found that prediction accuracy of the TensorFlow-based neuralnet and RF models were improved to 97% (authors stated no overfitting of the neural network model with TensorFlow from Keras) with the addition of augmented images [17]. They also found that unsupervised ML tends to incorrectly classify roots into a normal distribution and predicting most lines as the intermediate type and that by coupling root type with its prediction probability will give breeders a confidence level to make better selection decisions for breeding efforts by advancing the best or excluding the worst lines [17].

Another high-throughput pipeline was developed using deep neural network (DNN) models and automated feature extraction for field root phenotyping by Bauer et al. [94]. They applied their pipeline that used RootPainter DL segmentation to a small sample of 36,500 field-derived images of winter wheat roots and maize roots acquired from Bartz minirhizotron (RGB) and Vienna Scientific Instruments GmbH minirhizotron (RGB) scanners and then used RhizoVision Explorer to automatically perform feature extraction from the segmented images. They note that their pipeline outperformed manual annotation by 98.1% to 99.6% in terms of processing time and, by substantially reducing the processing time for minirhizotron imagery, has removed the image analysis bottleneck to high-throughput phenotyping approaches [94]. Huang et al. [35] developed a method to automatically and accurately segment high-resolution minirhizotron images. They investigated cotton roots using 92 original field-derived images (expanded to 552 using image augmentation) acquired from minirhizotrons (RGB). They automatically segmented roots using an improved/modified OCRNet model that included a global attention mechanism (GAM), which they called OCRNet + GAM. The improved model outperformed all the other tested models (FCN, PSPNet, DeepLabv3+, and OCRNet) in terms of accuracy (0.9866), recall (0.9419), F_1_ score (0.9146), and IoU (0.8426). By adding a GAM, the OCRNet model is forced to focus on the features of targets (roots) to improve the model’s ability to distinguish roots from the background (soil) and image noise (stones, worms, soil cracks, etc.)

RhizoVision Crown is backlit camera-based system [45] to acquire root crown imagery from the field that is easy to segment and was used by Mattupalli et al. [65] to image alfalfa root crowns from within and outside disease rings. In the original paper, linear discriminant analysis was used to predict status with 76% accuracy. A different research group used the publicly available imagery to classify on the basis of the WCSMO (water cycle spider monkey optimization)-based deep CNN [33]. They used their model to analyze and classify 264 monochrome camera images of alfalfa and cotton root rot. The model achieved higher sensitivity, specificity, and accuracy scores (91.69%, 92%, and 91.98% respectively) over the other existing models they compared it to (PA-RPL + DL, EEG routing + DL, Hybrid routing + Canny and Ostu, and PriNergy + ANN) [33]. This highlights the importance of sharing imaging data and methods for both reproducibility and reuse. An iterative neural network architecture approach to image segmentation of thin and reticulated root structures to identify root disease was created called ITErRoot [73]. Several ML models using several different genus seedling root rot types including cucumber (*Cucumis sativus* L.), rapeseed, soybean, and winter wheat from 1,278 laboratory-derived DSLR camera (RGB) images were tested. The ITErRoot model (based on IterNet [95] and the U-Net architecture) outperformed the other tested models (SegRoot [96] and IterNet) with quality metrics of mean DSC = 0.889, mean IoU = 0.807, sensitivity = 0.930, and specificity = 0.998. ITErRoot was a significant improvement over other recent approaches to root segmentation and performed particularly well with the presence of non-root objects such as disease [73]. Pierz et al. [97] developed a pipeline called RootDS that uses PlantCV [98] as its backbone to test common bean (*Phaseolus vulgaris* L.) roots and root rot caused by *Fusarium solani* species complex by assessing disease severity using 114 laboratory-derived images acquired from a photo scanner (RGB) [97]. They compared automated disease scores and root areas using PlantCV to manual researcher disease and area scoring and found a high correlation between both methods (*R*^2^ for PlanCV and manual methods = 0.92 for disease score and 0.90 for root area). The RootDS pipeline provides greater functionality in disease score datasets and provides an alternative for generating image sets for use in available Root System Markup Language (RSML) software [97].

Wang et al. [99] proposed a detection model modified from the YOLOv5 (released by Ultranytics) architecture, called YOLOv5-CMS, which they used to investigate cucumber root knots from 391 laboratory-derived images acquired from a DSLR camera (RGB). Their YOLOv5-CMS model enhanced the existing YOLOv5 model via a dual attention module (CBAM-CA) to detect root knots in an effort to support breeding nematode-resistant cucumber varieties. Their model was an improvement (of recall and mean average of precision) to the original YOLOv5 model of 3% and 3.1%, respectively. Quality metrics for the YOLOv5-CMS model were precision = 0.943, recall (sensitivity) = 0.885, F_1_ score = 0.913, and mean average of precision = 0.948. This improved model achieved higher quality metric values than all the other competing models (8 other models total), and it provides an effective method for obtaining more intuitive and accurate data sources during the breeding of cucumber varieties resistant to root-knot nematode [99].

A fully automated GUI-based tool for root segmentation and quantification using a pretrained CNN architecture capable of being utilized by unskilled users was developed for maize [37]. The model was trained using masks (6,465) created from 182 laboratory-derived images of maize roots acquired from a monochrome camera (near-infrared). Narisetti et al. [37] developed a model, called faRIA (based on U-Net model framework), that achieved a DSC accuracy of 0.87, thereby outperforming other existing models such as SegRoot (DSC of 0.67) and demonstrating that their framework allows the automatic and efficient analysis of soil–root interactions without manual interaction or parameter tuning. Smith et al. [43] also created a GUI, called RootPainter, for rapid training of DNNs for biological image analysis that allows corrective annotations through user-informed corrections to AI (U-Net-based variant) segmentation. They evaluated their software by investigating chicory roots using 300 laboratory-derived images acquired from a photo scanner (RGB). The model reported an average dice score for roots of 0.9, indicating that the model was accurate and comparable to previous results from Smith et al. [43,67]. Smith et al. [43] note that on the basis of their results, the DL models could be trained to high accuracy with several datasets of varying target objects, backgrounds, and image quality in as few as 2-h annotation time and that annotation, training, and data processing of numerous datasets can be achieved within a single day when using RootPainter. To test this finding, RootPainter was used by another team interested in measuring continuous root growth and investigated humidity and soil nitrogen effects on Norway spruce (*Picea abies* L.) sapling roots using 2,288 laboratory-derived images acquired from smartphone (Samsung S6 Edge and Samsung Galaxy S8+) cameras (RGB) [100]. When the model was used, a small reduction in F_1_ score occurred (0.88) when comparing model outputs to manual annotation of fine roots. Sell et al. [100] found that increased humidity reduced fine root growth and diminished sequential developmental peaks.

Griffiths et al. [101] was interested in the 4D characterization of RSA and the adaptive responses of field pennycress (*Thlaspi arvense* L.) RSA and development under varying nitrate regimes. They conducted their study using 72 laboratory-derived images created with a machine vision camera and extracted root traits using a GiARoots and DynamicRoots pipeline, while shoot traits such as rosette size and leaf counts were determined using a modified PlantCV pipeline. There were 3 root plasticity responses to nitrate treatments: (a) normal root development under sufficient or excess nitrate availability, (b) intense root foraging in response to limited nitrate, and (c) arrested root growth under critically insufficient nitrate conditions [101].

The above case studies elucidate the growing popularity of AI methods included in modern RSA research. A general trend seen in many of these studies is the pairing of semi- to fully automatic RSA trait extraction methods from a variety of newly developed software with the ever-increasing number and quality of AI/ML/DL models to analyze the extracted traits. Other important improvements include reduced manual and eyes-on labor requirements; increasing the size limits of manageable data (high-resolution and multichannel images); capably dealing with soil–root signatures in low-contrast, complex, or noisy background images; accurately classifying and segmenting primary and lower-order growth; and increasing the speed and training of models and the accuracy of their outputs via processes such as corrective annotations. In addition, some researchers have begun to bridge the gap between laboratory/greenhouse and field-based root phenotyping with more mobile, self-contained imaging platforms and/or growth observation apparatuses (such as root towers). Last, the frontier of 4D image analysis of RSA focused on root system formation, root growth such as foraging, and root development or senescence seems to be coming more into the forefront of research as technology is now able to keep pace with researchers’ desires to perform in situ, real-time analysis on RSA. In addition, the accuracies reported by individual authors/case studies are not consistent in that some authors use different metrics state their results. Without having the original data (confusion matrices) for every case study described in this review, there is (unfortunately) no way to standardize which quality metric (F scores, IoU, DSCs, etc.) is used to compare these studies.

From model development, root image analysis can be grouped into 2 phases based on the above case studies, phases I and II. The phase I RSA image analysis and ML process includes 2 steps: step 1, to extract pre-defined features from the images; and step 2, to use the extracted features (typically comma-separated value or RSML files) as predictions to apply ML prediction. Examples of research that utilize phase I methods are numerous and a variety of (some aforementioned) software have been developed specifically to extract root traits from imagery in this 2-step process, including (but not limited to) ARIA [102], DIRT [103], GLO-Roots [104], WinRhizo [105], and RhizoVision Explorer. Phase II RSA image analysis is just a one-step (typically automated as in Narisetti et al. [37]) analysis without extracting predefined features and using the images as the direct input to perform DL via CNN. Conversely, phase I RSA analysis uses a very small number of features as predictors for ML, whereas CV-based phase II image analysis uses all pixels from images, with thousands of pixel features with multiple layers of neural network for DL. The current state of the art in RSA image analyses can be described as phase II, with the caveat that there is still room for improvement on this concept/technique in that even at phase II, there is no analysis pipeline that is completely seamless (workflow steps featured in Fig. 2 that are combined in one fluid process) and fully automated or that captures the unharvested 3D root system of a plant in its entirety through time (4D).

## Discussion and Future Research

The concealed nature of roots by soil, which have limited researcher’s methods to destructive and/or techniques that exert laboratory controls on plant growth, is the main difficulty with studying plant roots and their functions, growth, and structure(s). These issues have not prevented scientists from digging up roots for RSA research. However, the main problem for RSA investigation/understanding is the lack of viewing entire root system structures through time in its natural growth environment.

Modern imaging technologies and novel sensors are now paving the way for more detailed yet less intrusive means of data collection that is allowing for more accurate portrayals of RSA and root research that was not possible in the recent past. Currently, investigative techniques are still mostly limited to RGB cameras and low-cost sensors not capable of looking through soil, with some notable exceptions such as tomography. However, high-resolution RGB imaging techniques coupled with the speed and accuracy of robotic automated processes and AI such as ML are pushing RSA research further into the realm of nondestructive, 4D, real-time imaging and analyses on entire root systems, individual traits of those systems, and the genetics or environments that drive them. Because of these advances, RSA research does have a promising path forward through ML imagery analyses capable of supplanting the somewhat former issue of soil opacity using scanning technologies that can aptly detect soil versus plant signatures. This new perceptive advantage in detection now ushers in researchers aimed at root/shoot associations that will likely drive the much discussed “Second Green Revolution” that many plant scientists see as the way forward and away from worsening global food security problems. Although still fledgling, RSA research paired with ML technology has already proven to be advantageous, and current advancements foreshadow new understandings of desired plant traits, such as increased yields from seemingly plateaued genetics via RSA improvements. In addition, modern hardware such as unmanned aerial vehicles (drones) and terrestrial robotics paired with ML technology offer automated management and/or commercial solutions to agricultural problems such as early identification and targeted intervention of diseases, pests, water or nutrient needs, as well as the ability to reduce costs and required management outputs such as water, pesticide, and herbicide applications, weed removal, crop assessment [in situ root growth, biomass, maturity, yields, etc.), and the actual harvesting or planting of crops (especially double-cropping in mature stands, such as cover crops like alfalfa within maize or Kernza (intermediate wheatgrass) rows].

Current progress and major advances in RSA research (at least for the time being) are coming from research focused on the areas of 2D and 3D imaging that rely on time-series analyses of data by AI techniques. As ML modeling improves by increasing the accuracy of models and their outputs, quicker, more mobile, and higher-resolution (spatial, temporal, spectral, and radiometric) analyses will likely follow. In addition, the expansion of ML to applications throughout the realm of agriculture will also undoubtedly propel the subdiscipline of cyber/digital agriculture forward as it proliferates into new lines of inquiry, such as the advances of ML and CV research into land cover classification, crop phenology, weed detection, and fruit grading. More approaches adopting long short-term memory or other RNN models that exploit the time dimension to achieve higher performance prediction and classification tasks such as yield prediction, water requirements, and the estimation of plant growth based on previous observations are expected in the future [106]. The value of studies located in the laboratory or greenhouse will also be exemplified once demonstrated in the field [107], and a plethora of recent studies indicate that the toolset of image analysis using ML helps to partially bridge the gap between laboratory/greenhouse and field experiments by augmenting traditional studies with faster, more accurate, nondestructive methods that are transformational to RSA inquiries and offer paradigm-shifting breakthroughs in the future [86]. The in silico analyses of “big” and eventually “mega” datasets that are also dense in terms of resolutions and holistic in nature (environmental, chemical, physiological, etc.) are likely to provide insights into plant growth and/or habits that are to-date unobserved, similarly to the way that seemingly unrelated spatial phenomena can show functional associations via the use of geospatial analyses in geographical information systems [108]. Last, we agree with Singh et al. [41] in that the integration of ML data analyses, collection, and curation into a cohesive and dovetailed pipeline will lead to large-scale applications of ML that have great potential to accelerate breeding efforts and predictive phenomics as outlined by the functional phenomics approach [109].

While there is no single method currently sufficient to completely understand the complexities of RSA of any one plant species and/or RSA’s interplay with the environment and conditions that govern plant growth, many currently used methods offer strengths and the promise of continuing to partially disentangle the controls on RSA and plant roots and the subsequent effects/controls of RSA on other plant organs and plant traits. Future research that observes, quantifies, explains, and predicts incremental growth, growth habits, and the variables and/or environmental conditions that control these are still needed to better understand the true nature of RSA and its role(s) and influences on traits desired by plant breeders and geneticists. Because the determinants of RSA for most species/cultivars of plants are still not fully understood, the many covariates that need to be disentangled to understand whether observed phene states are expressions of genetics or environmental conditions such as climate, edaphic controls, allelopathy, or other influences such as disease and/or pests need to be investigated further. Major challenges for the future appear to be tied to hard-won and time-limiting genetics research, the limitations of technology (speed, processing ability, and skilled laborer ability), the often-limited resolutions of data (such as ground-penetrating radar), the need for sufficient image dataset sizes capable of overcoming parameterization issues such as overfitting, adequate signal-to-noise ratios (a greater problem for some sensor types such as minirhizotrons), and the high cost and nonportability of the most advanced imaging systems such as MRI and other tomographic scanners. In addition, data quality issues are also major current (and future) challenges for RSA researchers who will require concerted efforts to either remedy and/or prevent the deleterious effects of “bad” or “tainted” data into the research stream.

Tardieu et al. [110] discuss the “Big Data” challenge of plant phenomics, which involves combining the datasets and methods from disparate phenotyping platforms to perform meta-analysis using large-scale association genetics that could potentially yield results for country or continental levels. This level of aggregation of experiments/accessions would require a minimum of some standardization of hardware, software, and methods to create meaningful and robust results. Of course, there is no governing body to mandate any standard in this context; however, interested parties and stakeholders could be compelled to enter into an agreement to standardize their methods so to make their individual scientific contributions congruent with others interested in the same goal. In some ways, steps toward this have already been taken, for example, RSML was advanced by Lobet et al. [111], has been adopted by several researchers, and is widely supported by the community according to Yasrab et al. [87].

Phase II analyses in which root images are used as direct inputs into automated DL models that are capable of segmenting, classifying, and predicting RSA appear to be the next wave of RSA research based on the observed trends discussed herein. As the past is prologue, much imagery-based analyses are borrowed or carried over from the medical sciences, and the modern trend in AI-driven imagery research in those fields has naturally bled into the plant sciences because of the diffusion of proven techniques there and in other fields such as self-driving vehicle engineering. In addition to images as direct inputs to models, the apparent trend of in situ, 4D, and/or real-time data collection and analyses is being undertaken by many researchers, although the portability, background noise caused by soil artifacts, and the high costs of some sensors such as x-ray CT and MRI scanners are major limiting factors to this research. We believe that phase II RSA image analysis research has yet to see its zenith, and when the preprocessing steps such as segmentation and trait/feature labeling and recognition are fully automated within the framework of DL models, a new phase and frontier of RSA research will be reared (phase III). We speculate that in a phase III future, the state of the art in RSA image analysis will involve AI models that only require raw, real-time, and in situ images as input and that can leverage millions of vetted RSA/plant images in a standardized, public, benchmarking library of images to train/test/validate/predict the content of that single input image (or video, 4D). The benefits of standardized methods, data, and sharing/dissemination between scientists are known (science and literature are examples of this) so it seems logical (albeit far reaching) to assume that a natural outcome of our efforts regarding RSA would eventually produce a repository of data and methods that eventually could identify, diagnose, and/or predict a host of currently out of reach information from an unlabeled image (or series, 4D) of a single plant’s RSA such as its genetics or phenomics, environmental conditions, or stresses that it grew in (soil, climate, disease, pests, etc.). The fact that one can add an image, song, or block of text to some popular search engines driven by AI that produces meaningful, thorough, and descriptive outputs about the input is a perfect example of the possibility of a phase III RSA image analysis future.

In this paper, we have discussed how the applications, intersections, and importance of RSA research, imaging techniques, and ML technologies have advanced plant root studies. Past research indicates that while RSA research has made significant progress in the past decade, some of the major limitations that still exist and continue to challenge researchers are the high costs and the lack of portability of some imaging sensors and platforms into the field allowing for fully in situ, real-time, automated analyses. We believe that the future for advanced imaging technology coupled with improved ML techniques and models is promising and that much progress is on the way, given the recent major achievements in ML and CV modeling that is growing in accuracy, predictive power, and computational speed as researchers drill down on these hurdles in their own studies.
